# PARP Inhibitors in Patients With Newly Diagnosed Advanced Ovarian Cancer: A Meta-Analysis of Randomized Clinical Trials

**DOI:** 10.3389/fonc.2020.01204

**Published:** 2020-08-04

**Authors:** Yizi Wang, Fang Ren, Zixuan Song, Xiaoying Wang, Chiyuan Zhang, Ling Ouyang

**Affiliations:** Department of Obstetrics and Gynecology, Shengjing Hospital of China Medical University, Shenyang, China

**Keywords:** newly diagnosed advanced ovarian cancer, PARP inhibitors, homologous recombination deficiency, BRCA mutation, meta-analysis

## Abstract

**Background:** The efficacy of poly(adenosine diphosphate–ribose) polymerase inhibitors (PARPi) as a maintenance therapy in patients with newly diagnosed advanced ovarian cancer remains unclear. We conducted a meta-analysis to assess the benefits and safety of PARPi maintenance therapy in patients with newly diagnosed advanced ovarian cancer.

**Methods:** We searched the PubMed, EMBASE, and Cochrane databases for randomized controlled trials (RCTs), which assessed the efficacy of PARPi as a maintenance therapy for newly diagnosed advanced ovarian cancer. Progression-free survival (PFS) was the primary endpoint, which was assessed using hazard ratios (HRs) with 95% confidence intervals (95% CI). Progression-free survival was extracted independently, and the pooled results were used to compare the prognoses of patients who received PARPi maintenance therapy and those who received a placebo.

**Results:** Three RCTs, SOLO1, VELIA/GOG-3005, and PRIMA, which included 1,881 patients with newly diagnosed advanced ovarian cancer, were included in the meta-analysis. The overall analysis showed that PARPi maintenance therapy significantly increased PFS (HR, 0.51; 95% CI, 0.33–0.80; *P* = 0.004) compared to placebo. Subgroup analyses confirmed this result. We also observed an improved PFS in patients with homologous recombination deficiency (HR, 0.50; 95% CI, 0.38–0.66; *P* < 0.001) and in patients with BRCA mutations (HR, 0.42; 95% CI, 0.31–0.57; *P* < 0.001). Moreover, there were no significant differences in health-related quality of life between the PARPi and placebo groups.

**Conclusions:** Patients with newly diagnosed advanced ovarian cancer who received PARPi maintenance therapy had a better prognosis than did those who received a placebo. Moreover, no significant changes in health-related quality of life were seen in PARPi-treated individuals.

## Introduction

Ovarian cancer is the most lethal gynecological malignancy (1, 2). There were ~22,000 new cases and 14,000 deaths due to ovarian cancer during 2019 in the United States (3). More than 70% of ovarian cancer patients are diagnosed in the advanced stage (4). Currently, the primary treatment for newly diagnosed advanced ovarian cancer is a combination of optimal debulking surgery and platinum/taxane-based chemotherapies (5). Unfortunately, the majority of patients with advanced ovarian cancer will have a recurrence within 3 years (6).

Targeted therapies are a new treatment option for patients with ovarian cancer (7). Poly(adenosine diphosphate–ribose) polymerase (PARP) can prevent DNA repair in tumors with homologous recombination deficiency (HRD), including those with BRCA1 or BRCA2 mutations (8). Approximately 13% of ovarian cancers are caused by a mutation in BRCA1 or BRCA2 (9). Poly(adenosine diphosphate–ribose) polymerase inhibitors (PARPi) including niraparib, rucaparib, and olaparib have been approved as maintenance therapies for relapsed platinum-sensitive ovarian cancer patients regardless of BRCA status (10). However, it is unclear if PARPi can improve the prognosis of patients with newly diagnosed advanced ovarian cancer.

Recently, results from two separate phase 3, multicenter, randomized trials of PARPi in patients with newly diagnosed advanced ovarian cancer were published, and they both found that PARPi could improve the progression-free survival (PFS) of patients with newly diagnosed advanced ovarian cancer when PARPi were used as a maintenance therapy (11, 12). Therefore, we performed a meta-analysis to assess the efficacy of PARPi maintenance treatment for patients with newly diagnosed advanced ovarian cancer.

## Methods

### Search Strategy and Data Sources

A comprehensive search of clinical trials published before December 1, 2019, in the PubMed, EMBASE, and Cochrane databases was performed in accordance with PRISMA (Preferred Reporting Items for Systematic Reviews and Meta-Analyses) guidelines (Supplementary Table 1). The following search terms were used: “poly(ADP-ribose) polymerase inhibitors,” “inhibitors of poly(ADP-ribose) polymerases,” “poly(ADP-ribosylation) inhibitors,” “PARP inhibitors,” “inhibitors, PARP,” “olaparib,” “niraparib,” “veliparib,” “rucaparib,” and “ovarian neoplasm,” “ovarian cancer,” “cancer of ovary.” There were no restrictions with regard to language. The references in the selected studies were also scrutinized to further identify relevant studies.

### Inclusion and Exclusion Criteria

PICOS (population, intervention, comparison, outcomes, and study design) guidelines were used to formulate inclusion and exclusion criteria. The inclusion criteria were as follows: (1) population: patients with newly diagnosed high-grade serous or endometrioid ovarian cancer of FIGO (International Federation of Gynecology and Obstetrics) stage III or IV; (2) intervention: PARPi were used as a maintenance treatment; (3) comparison: patients receiving oral PARPi as a maintenance treatment vs. patients receiving a placebo; (4) outcomes: PFS was compared between the PARPi group and the placebo group; and (5) study design: randomized controlled trials (RCTs).

The exclusion criteria were as follows: (1) Population: patients with relapsed ovarian cancer and patients with bevacizumab as maintenance treatment in first line; (2) Intervention: patients did not receive oral PARPi as maintenance treatment; (3) Comparison: there were no control or placebo groups; (4) Outcomes: studies without PFS measurements; (5) Study design: studies that were not RCTs.

### Data Extraction and Study Quality Assessment

Two reviewers independently reviewed the included studies and extracted the following data: first author, year of publication, trial acronym, study period, follow-up time, number of total patients enrolled, FIGO stage, and PFS. The risk of bias approach proposed by the Cochrane Collaboration (13) was used to assess the quality of the included RCTs. Any discrepancies were discussed among all authors and identified by consensus.

### Statistical Analysis

The primary endpoint of this meta-analysis was PFS, which was assessed using hazard ratios (HRs). Stata software, version 12.0 (2011; Stata Corp., College Station, TX, USA), was used to perform the meta-analysis. Hazard ratios are presented with 95% confidence intervals (CIs). A random-effects model was used in all analyses. Significant two-tailed *P* < 0.05 was considered significant. We used Cochran's Q test and the *I*^2^ statistic to evaluate the heterogeneity among the studies (14, 15). The robustness of the results was assessed using sensitivity analyses (16). Subgroup analyses were conducted based on age, FIGO stage, the timing of chemotherapy in relation to surgery, BRCA mutation status, and homologous recombination status. Funnel plots that are used to assess publication bias were not performed for the limited number of included studies.

## Results

### Study Selection

**Figure 1:**
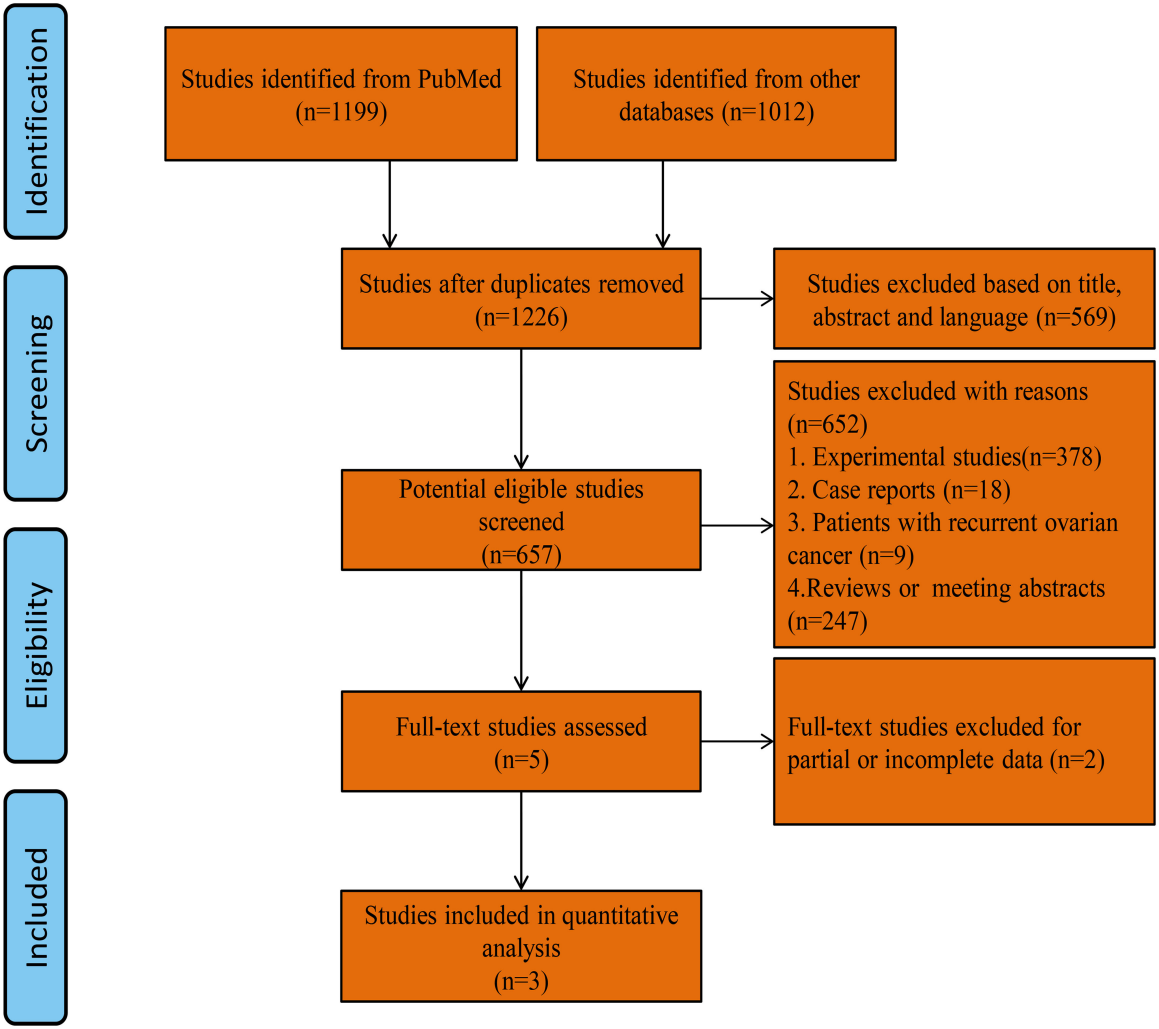
Flow diagram of trial selection.

**Figure 2:**
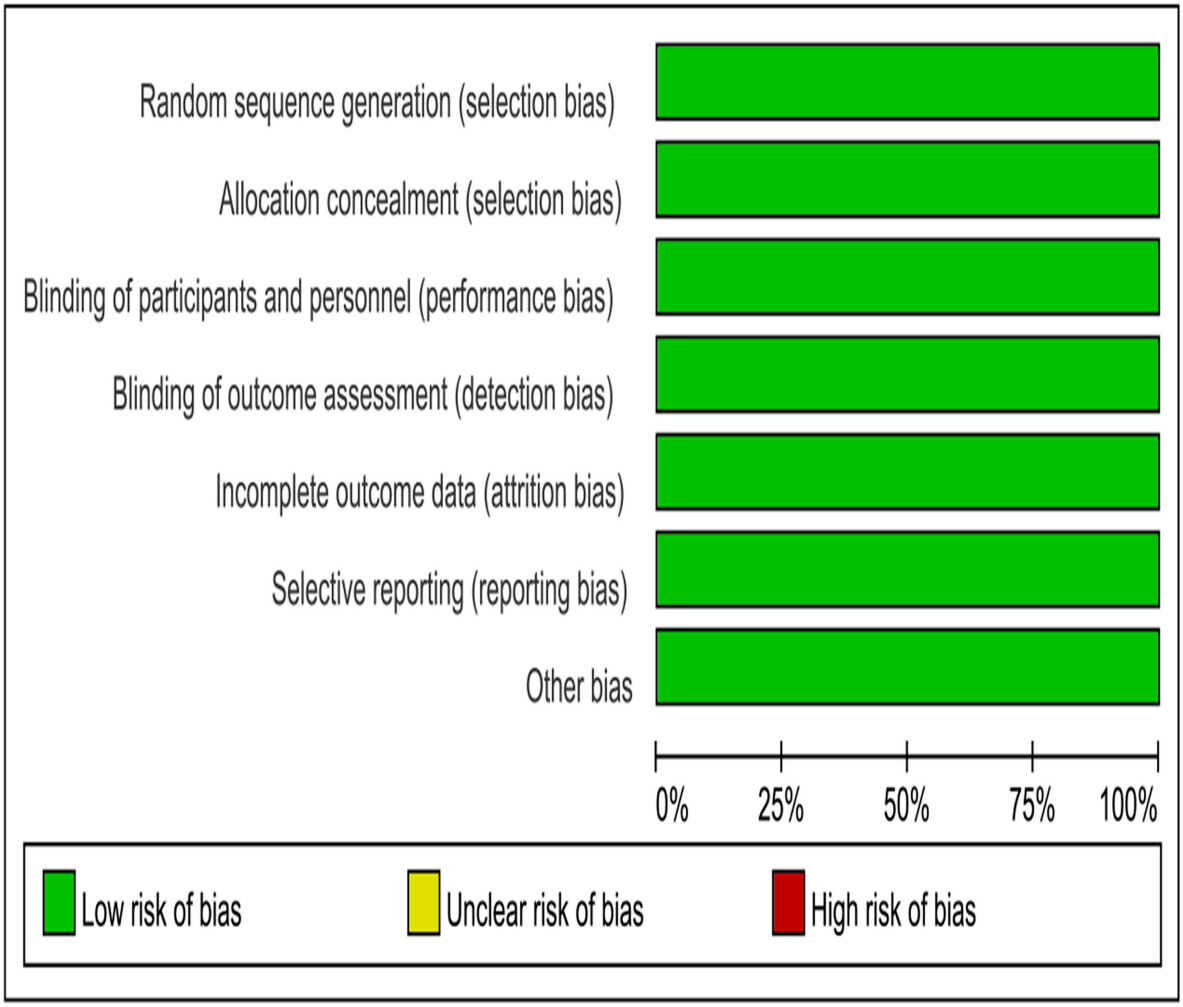
Risk-of-bias graph.

A total of 1,226 studies were identified using our search strategy. After screening of the abstracts or titles, the full texts of five studies were further reviewed. Three RCTs that met the study inclusion criteria were selected for analysis (PARPi group = 1,129, placebo group = 752; total = 1,881 patients): VELIA/GOG-3005 (PARPi group = 382, placebo group = 375; total = 757 patients), PRIMA (PARPi group = 487, placebo group = 246; total = 733 patients), and SOLO1 (PARPi group = 260, placebo group = 131; total = 391 patients) (11, 12, 17). A flow diagram of the trial selection is illustrated in Figure 1. The quality of the RCTs was evaluated using the “risk of bias” tool according to the *Cochrane Handbook* (Figure 2). The main characteristics of the population involved in the studies are represented in Table 1.

**Table 1:** Main characteristics of the study populations in the included studies.

**Study**	**Trial acronym**	**Medication**	**Study period**	**Follow-up (median months)**	**Total patients**	**Age at baseline**		**Stage**		**PARPi**	**Placebo**	**BRCA mutation**		**HRD**	
						**<65 year**	**>65 year**	**III**	**IV**			**PARPi**	**Placebo**	**PARPi**	**Placebo**
Coleman et al. (12)	VELIA/GOG-3005	Veliparib	2015–2017	28	757	461	296	587	169	382	375	108	92	214	207
González-Martín et al. (11)	PRIMA	Niraparib	2016–2018	13.8	733	444	289	476	257	487	246	152	71	169	80
Moore et al. (17)	SOLO1	Olaparib	2013–2015	40.7	391	337	54	325	66	260	131	260	NA	131	NA

*PARPi, poly(adenosine diphosphate–ribose) polymerase inhibitors; HRD, homologous recombination deficiency; NA, not available*.

### PARPi vs. Placebos for Ovarian Cancer Patients

**Figure 3:**
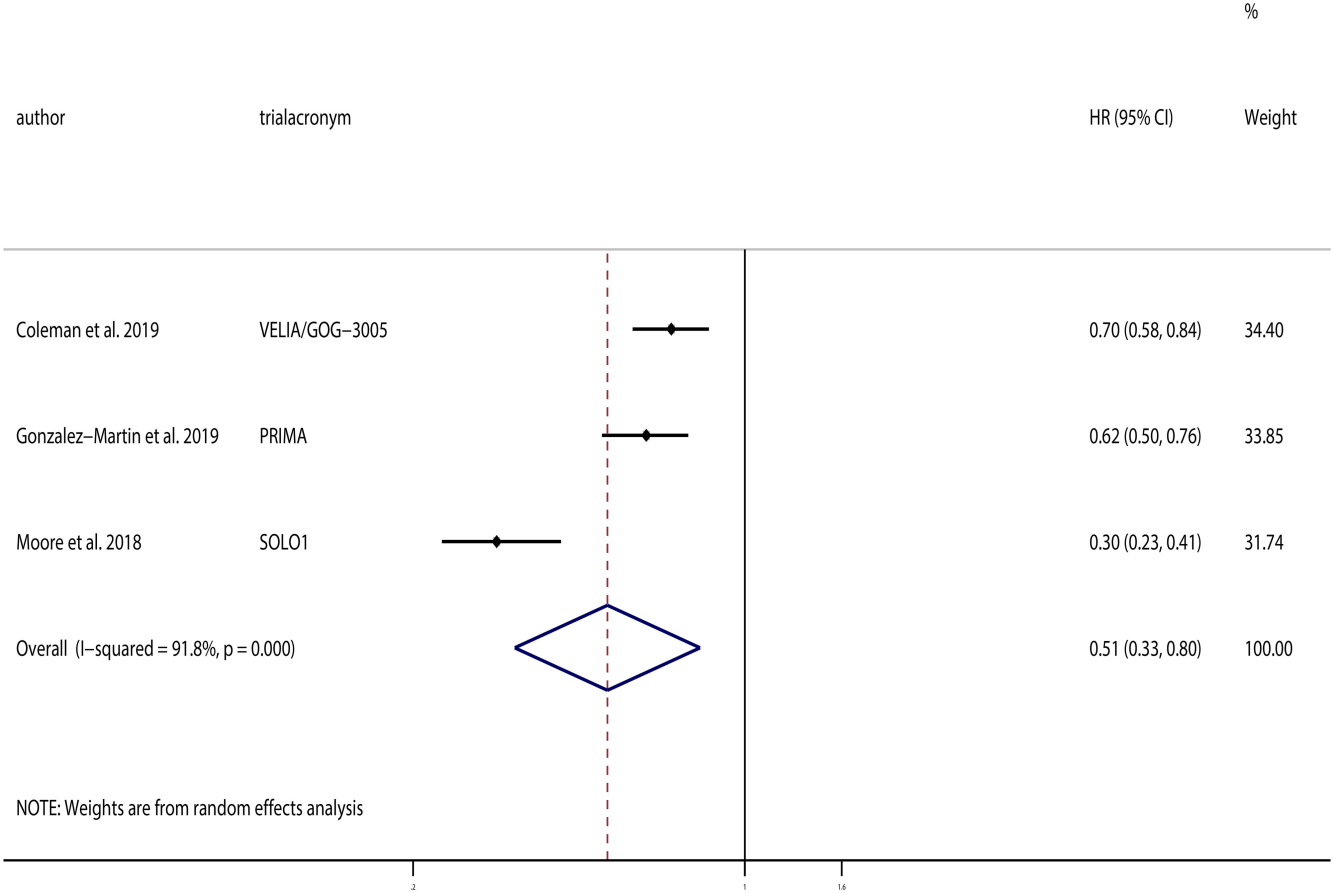
Progression-free survival (PFS) of all patients.

All three of the selected trials provided PFS data. The pooled analysis indicated that PARPi maintenance treatment could significantly improve PFS compared to the placebos (HR, 0.51; 95% CI, 0.33–0.80; *P* = 0.004; Figure 3). Although substantial heterogeneity existed (χ^2^ = 24.29; *P* < 0.01, *I*^2^ = 91.8%), sensitivity analyses were conducted, which demonstrated that the result was robust.

#### Which Ovarian Cancer Patients Could Benefit From PARPi?

We conducted a subgroup analysis based on age, comparing PFS in patients categorized as <65 and >65 years of age. There were 1,242 patients <65 years old and 639 patients >65 years old. And we found that PARPi improved PFS in both groups (<65 years: HR, 0.51; 95% CI, 0.34–0.76; *P* = 0.001; >65 years: HR, 0.61; 95% CI, 0.45–0.83; *P* = 0.002).

All three trials conducted separate analyses of patients with FIGO stage III and IV ovarian cancer. There were 1,388 patients of stage III and 492 patients of stage IV included. Subgroup analysis based on the FIGO stage showed that PARPi improved PFS in both stage III ovarian cancer patients (HR, 0.49; 95% CI, 0.33–0.74; *P* = 0.001) and stage IV patients (HR, 0.74; 95% CI, 0.58–0.94; *P* = 0.016) compared to patients receiving placebos.

Patients in all three trials received PARPi as a maintenance therapy, whereas VELIA/GOG-3005 added PARPi to first-line chemotherapy. Subgroup analysis of the other two studies that did not combine PARPi with chemotherapy demonstrated a significant improvement in PFS in PARPi group compared to the control group (HR, 0.43; 95% CI, 0.21–0.88; *P* = 0.022).

VELIA/GOG-3005 and PRIMA used PARPi in patients who underwent interval surgery and primary surgery. We performed subgroup analysis based on the timing of chemotherapy in relation to surgery, and we found that PARPi improved PFS in both the interval surgery group (HR, 0.61; 95% CI, 0.50–0.74; *P* < 0.001) and the primary surgery group (HR, 0.70; 95% CI, 0.57–0.86; *P* < 0.001).

**Figure 4:**
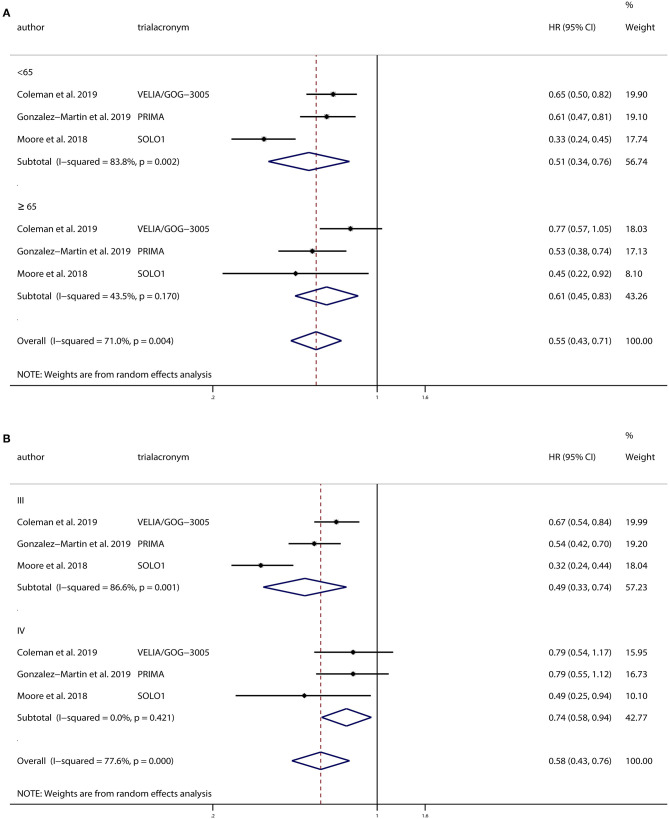
Progression-free survival (PFS) of **(A)** subgroups based on age and **(B)** subgroups based on International Federation of Gynecology and Obstetrics stage.

Additionally, we performed subgroup analyses based on BRCA mutation and homologous recombination status. VELIA/GOG-3005 and PRIMA analyzed PARPi use in patients with or without BRCA mutations. Subgroup analyses based on BRCA mutation status were conducted, and the results indicated that PARPi significantly improved PFS in patients with BRCA mutations (HR, 0.42; 95% CI, 0.31–0.57; *P* < 0.001), but not in patients without BRCA mutations (HR, 0.67; 95% CI, 0.43–1.04; *P* = 0.077) compared to the placebo groups. In addition, VELIA/GOG-3005 and SOLO1 analyzed BRCA1 and BRCA2 separately. Hence, we conducted subgroup analyses of BRCA1 and BRCA2 patients and found that PARPi improved PFS in patients with BRCA1 mutations (HR, 0.39; 95% CI, 0.30–0.52; *P* < 0.001), but not in patients with BRCA2 mutations (HR, 0.35; 95% CI, 0.11–1.08; *P* = 0.067) compared to the placebo groups. Moreover, VELIA/GOG-3005 and PRIMA analyzed PARPi as a maintenance treatment in patients with HRD and homologous recombination proficiency. We also performed a subgroup analysis and found that PARPi maintenance therapy was associated with improved prognosis both in patients with HRD (HR, 0.50; 95% CI, 0.38–0.66; *P* < 0.001) and in patients with homologous recombination proficiency (HR, 0.75; 95% CI, 0.60–0.93; *P* = 0.010). The results from the subgroup analyses are illustrated in Figure 4.

### Systematic Review of Safety and Health-Related Quality of Life

**Figure 5:**
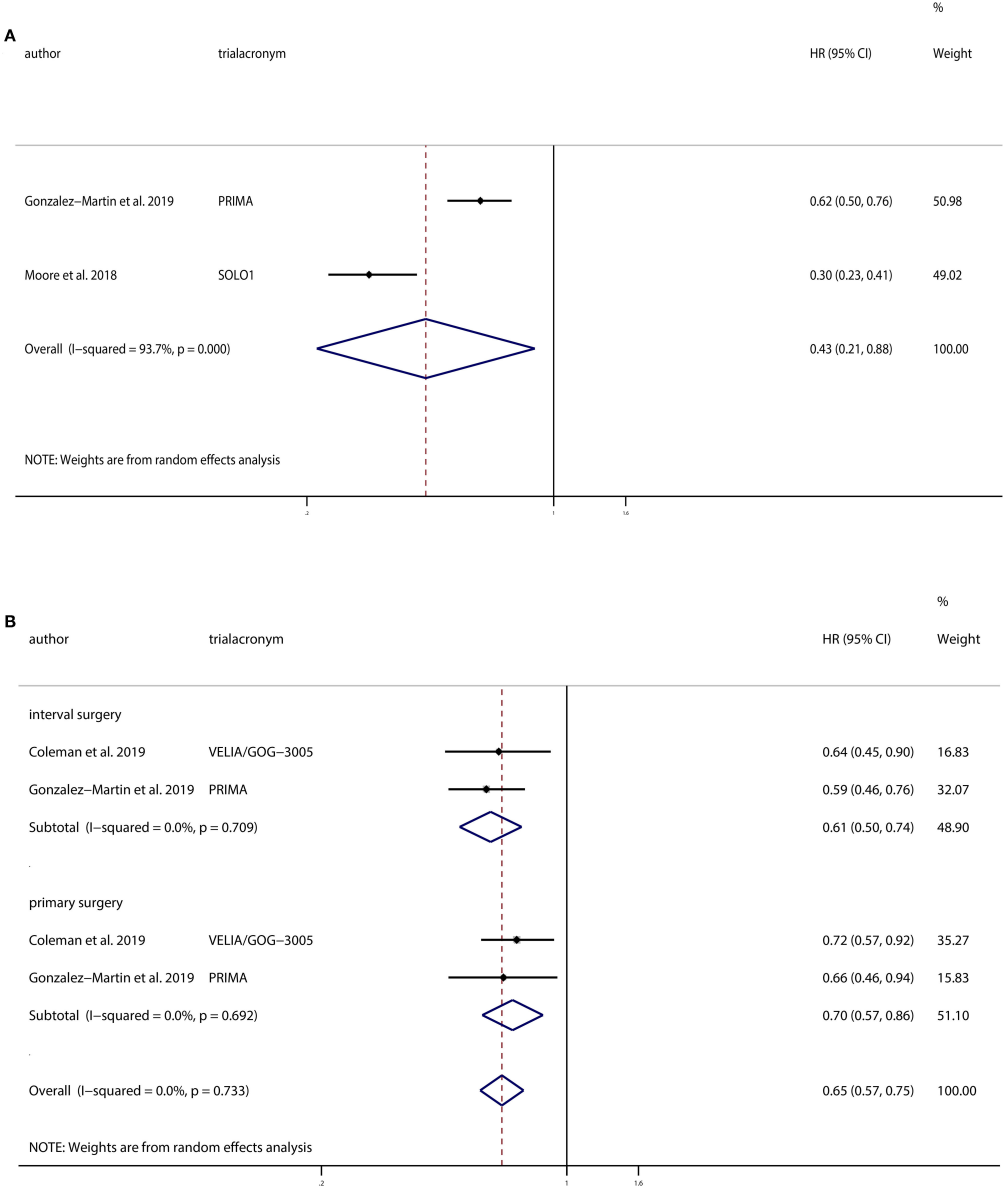
Progression-free survival (PFS) of **(A)** subgroup of patients without PARPi in combination with chemotherapy and **(B)** subgroups based on the timing of chemotherapy in relation to surgery.

**Figure 6:**
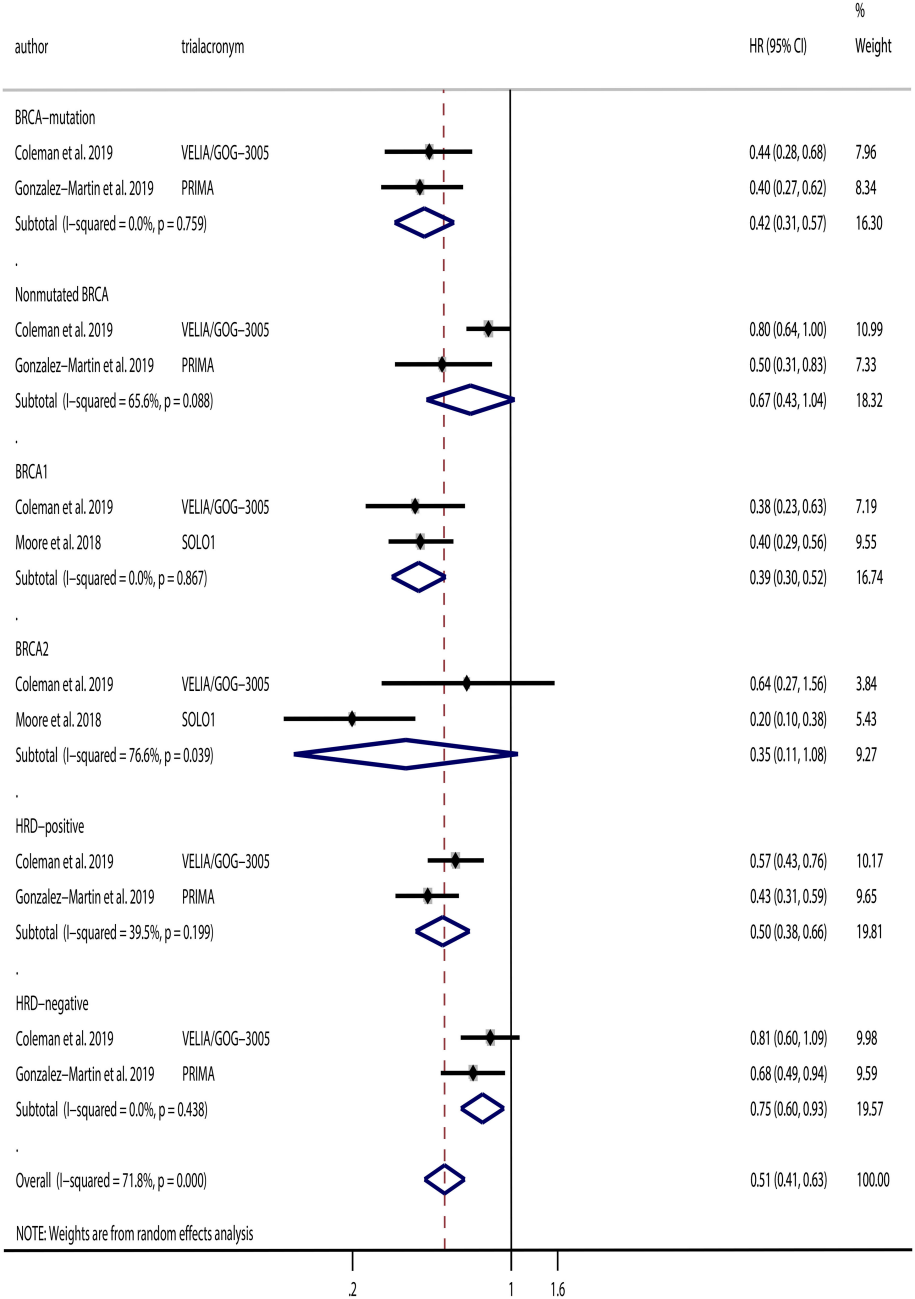
Progression-free survival (PFS) of subgroups based on BRCA mutation and homologous recombination status.

All the three RCTs investigated adverse events during the trials. In VELIA/GOG-3005 and SOLO1, most adverse events were grade 1 or 2, and the percentages of patients experiencing adverse events were similar in the PARPi group and the control group. Anemia was the most common serious adverse event in the SOLO1 trial, with 22% of patients presenting anemia grade 3 or more in the olaparib arm as compared to 2% in the placebo arm. And the main reasons for discontinuation of olaparib therapy were anemia (2.3%) or nausea (2.3%). In the VELIA/GOG-3005 trial, 28% of patients in the niraparib arm presented thrombocytopenia grade 3 or grade 4 compared to 8% in the control arm, and the main reason for discontinuation of veliparib therapy was nausea (8%). PRIMA reported that 70% of patients in the niraparib group had grade 3 or higher adverse events compared to 18.9% in the placebo group. The most common severe adverse event was hematological toxicity, which was also the most common reason for discontinuation. The proportion of discontinuation was 12.0% in the niraparib arm and 2.5% in the placebo arm. All three trials also assessed health-related quality of life, and they all found that there were no clinically significant differences between the PARPi and the control groups (Figures 5, 6).

## Discussion

Over the past decade, many trials of targeted agents have been conducted in order to improve prognosis of ovarian cancer (18), including vascular endothelial growth factor inhibitors (19) and bevacizumab maintenance therapy after first-line chemotherapy for advanced disease (2). Poly(adenosine diphosphate–ribose) polymerase inhibitors have been proven to be effective in patients with platinum-sensitive recurrent ovarian cancer regardless of BRCA and HRD status (10). However, studies examining PARPi maintenance treatment in patients with newly diagnosed ovarian cancer are limited.

Several trials have investigated PARPi use in relapsed ovarian cancer patients, and Tomao et al. (10) conducted a meta-analysis to assess the efficacy of PARPi in platinum-sensitive recurrent ovarian cancer. Since the first report of the olaparib maintenance therapy trial results in newly diagnosed advanced ovarian cancer patients in 2018 (17), physicians have begun to use veliparib and niraparib in newly diagnosed patients (11, 12). Therefore, it is important and timely to assess the benefits associated with PARPi maintenance therapy in newly diagnosed advanced-stage ovarian cancer patients.

Three studies (SOLO1, VELIA/GOG-3005, and PRIMA) with a combined total of 1,881 advanced ovarian cancer patients were included in this meta-analysis. Our pooled results showed that PARPi maintenance therapy could improve PFS of patients with newly diagnosed advanced ovarian cancer. Subgroup analyses based on age also demonstrated an improvement in PFS in patients both <65 and >65 years of age. We next performed subgroup analyses based on FIGO stage, and all the three trials indicated that patients with stage III ovarian cancer who received PARPi had an improved PFS compared to patients who received placebos. For stage IV patients, SOLO1 (17) found a significant improvement in PFS in the PARPi group, but VELIA/GOG-3005 (12) and PRIMA (11) did not find any differences between the PARPi and placebo groups. When we conducted the subgroup analyses based on FIGO stage, we found that PARPi maintenance therapy was associated with an improvement in PFS in stage III regardless BRCA mutation and stage IV in BRCA mutation alteration.

Poly(adenosine diphosphate–ribose) polymerase inhibitors have been shown to improve PFS when added to chemotherapy and followed as a maintenance treatment in recurrent ovarian cancer (20). Patients in VELIA/GOG-3005 received veliparib combined with chemotherapy, and then veliparib was used as maintenance treatment (12). Thus, we conducted a subgroup analysis of the other two studies that did not combine PARPi with chemotherapy, and we found that maintenance therapy with PARPi significantly improved PFS.

The use of neoadjuvant chemotherapy in advanced ovarian cancer continues to be debated. Although some studies have reported an inferior prognosis in patients with neoadjuvant chemotherapy compared to primary surgery (21, 22), a recent meta-analysis found no difference in overall survival (OS) or PFS between patients who underwent neoadjuvant chemotherapy or primary surgery (23). Poly(adenosine diphosphate–ribose) polymerase inhibitors have also been researched in patients undergoing both neoadjuvant chemotherapy and primary surgery. Our subgroup analysis based on the timing of chemotherapy in relation to surgery demonstrated that PARPi maintenance therapy was associated with an improved prognosis both in patients who underwent interval surgery and in those who underwent primary surgery.

Konstantinopoulos et al. (24) had reported that approximately half of epithelial ovarian cancers have defective repair pathways of homologous recombination including BRCA1/2 mutations. Seo et al. (25) found an improved PFS in BRCA2 mutation patients compared to BRCA2 wild-type patients, but this was not seen in patients with BRCA1 mutations. Poly(adenosine diphosphate–ribose) polymerase inhibitors exhibit greater therapeutic effects in patients with germline or somatic BRCA mutations than those with wild-type BRCA (26). Poly(adenosine diphosphate–ribose) polymerase inhibitors may cause tumor cell death through regulation of DNA repair in BRCA1/2 mutant–selected tumors (27). Previous RCTs have shown that BRCA-mutated patients with recurrent ovarian cancer could benefit from PARPi (28–31). The trials included in our analysis all tested the BRCA mutation status of ovarian cancer patients. A majority of patients (388 of the 391 patients) included in SOLO1 had germline BRCA mutations. SOLO1 observed an improved PFS both in patients with BRCA1 mutations and in patients with BRCA2 mutations. In addition, VELIA/GOG-3005 and SOLO1 provided comparisons of PFS between the PARPi and placebo groups in patients with BRCA1 and BRCA2 mutations separately. VELIA/GOG-3005 observed an improvement in PFS in patients with BRCA mutations and patients with BRCA1 mutations, but not in patients with BRCA2 mutations or without BRCA mutations. PRIMA observed an improved PFS in patients with niraparib maintenance therapy compared to placebo regardless of HRD status. However, VELIA/GOG-3005 observed an improvement in PFS only in patients with HRD. When we conducted subgroup analyses based on homologous recombination and BRCA mutation status, we found that PARPi significantly improved PFS in patients with BRCA mutations or HRD, particularly those with BRCA1 mutations. However, there were no differences between PARPi and placebos in patients with BRCA2 mutations or patients without BRCA mutations. In patients without HRD, we observed an improved PFS in the PARPi group, which seemed to be inconsistent. As the two trials (PRIMA and VELIA/GOG-3005) had different criteria for HRD and had different results, and the upper limit of the 95% CIs of our pooled results was ~1.00, we cannot confirm the clinical significance of these findings.

To the best of our knowledge, this was the first meta-analysis to explore PARPi maintenance therapy in newly diagnosed advanced ovarian cancer. This meta-analysis was conducted according to PRISMA, and we used PICOS to determine the inclusion criteria. The studies we included were all well-designed, high-quality RCTs.

However, some limitations in our meta-analysis should be stated. First, the heterogeneity of population among the included trials was significant. The tumor characteristics of patients enrolled in the three trials were not consistent. For example, SOLO1 observed the PFS of presence or not of residual tumor after debulking surgery, whereas VELIA/GOG-3005 observed them in primary surgery group and interval surgery group, respectively, and PRIMA did not specify presence or absence of residual tumor after surgery. Thus, we could not combine the results according to the presence or absence of residual tumor after surgery. Second, the heterogeneity of inclusion criteria and exclusion criteria was not consistent in different studies. Although PRIMA and VELIA/GOG-3005 tested the homologous recombination status, they used a different criterion to define HRD. These issues contribute to the heterogeneity of the meta-analysis. Third, the trials researched on the maintenance therapy with different drug (olaparib, niraparib, and veliparib). Fourth, we could not determine OS because of the lack of OS data in the RCTs, which may have provided a more convincing result. Fifth, the number of the studies included was limited, and more, larger and high-quality RCTs are needed to confirm our conclusion.

## Conclusion

Poly(adenosine diphosphate–ribose) polymerase inhibitor maintenance therapy may improve PFS in patients with newly diagnosed advanced ovarian cancer, especially patients with BRCA mutations or HRD regardless age, stage at diagnosis, and time to surgery performed. There were no clinically significant differences in health-related quality of life between the PARPi and placebo groups.

## Data Availability Statement

All datasets generated for this study are included in the article/Supplementary Material.

## Author Contributions

YW and LO designed the study idea and the study methodology. YW, ZS, and FR performed the research and analyzed the data. XW and CZ screened full texts and performed quantitative data analyzing. YW wrote the manuscript. All authors read and approved the version of the manuscript, contributed to the article, and approved the submitted version.

## Conflict of Interest

The authors declare that the research was conducted in the absence of any commercial or financial relationships that could be construed as a potential conflict of interest.
